# *Mesencephalic astrocyte-derived neurotrophic factor* reduces cell apoptosis via upregulating HSP70 in SHSY-5Y cells

**DOI:** 10.1186/s40035-017-0082-8

**Published:** 2017-05-22

**Authors:** Hui Sun, Ming Jiang, Xing Fu, Qiong Cai, Jingxing Zhang, Yanxin Yin, Jia Guo, Lihua Yu, Yun Jiang, Yigang Liu, Liang Feng, Zhiyu Nie, Jianmin Fang, Lingjing Jin

**Affiliations:** 1 Department of Neurology, Shanghai Tongji Hospital, Tongji University School of Medicine, 389 Xincun Road, Shanghai, 200065 People’s Republic of China; 2 School of Life Science and Technology, Tongji University, 1239 Siping Road, Shanghai, 200092 People’s Republic of China; 3 Biomedical Research Center, Tongji University Suzhou Institute, Building 2, 198 Jinfeng Road, Wuzhong District, Suzhou, Jiangsu 215101 China

**Keywords:** HSP70, MANF, Parkinson’s disease, SHSY-5Y cell

## Abstract

**Background:**

Mesencephalic astrocyte-derived neurotrophic factor (MANF) is a new candidate growth factor for dopaminergic neurons against endoplasmic reticulum stress (ER stress). HSP70 family, a chaperon like heat shock protein family, was proved to be involved in the MANF induced survival pathway in 6-OHDA treated SHSY-5Y cells. However, the ER stress relative transcriptome, in MANF signaling cascades is still investigated. The involvement of HSP70, a 70kd member of HSP70 family, need further to be verified.

**Methods:**

The cell apoptosis was assayed by MTT, TUNEL staining and western blot of cleaved Caspase-3. The differentially expressed genes in SHSY-5Y cells under different conditions (control, 6-OHDA, 6-OHDA + MANF) were investigated by RNA-seq. Expression of HSP70 was further confirmed by real-time PCR. RNAi knockdown for HSP70 was performed to investigate the role of HSP70 in the MANF signaling pathway.

**Results:**

MANF inhibits 6-OHDA-induced apoptosis in SHSY-5Y cells. Six ER stress relative genes (HSP70, GRP78, xbp-1, ATF-4, ATF-6, MAPK) were found enriched in 6-OHDA + MANF treatment group. HSP70 was the most significantly up-regulated gene under 6-OHDA + MANF treatment in SHSY-5Y cells. RNAi knockdown for HSP70 inhibits the protective effects of MANF against 6-OHDA toxicity in SHSY-5Y cells.

**Conclusion:**

MANF exerts a protective role against 6-OHDA induced apoptosis in SHSY-5Y cells via up-regulating some ER stress genes, including HSP70 family members. The HSP70 expression level plays a key role in MANF-mediated survival pathway.

## Background

Parkinson’s disease (PD) is one of the most common neurodegenerative disorders of the central nervous system. It is characterized by chronic and progressive loss of midbrain dopaminergic (DA) neurons. Over the past years, the precise etiology and disease pathogenesis are mostly unknown. Current therapy strategies aim at symptomatic relief rather than preventing disease progression. The use of neurotrophic factors represents a potential treatment strategy, which is essential to neuronal differentiation and maturation during development and adulthood.

The newest candidate growth factor for dopaminergic neurons is mesencephalic astrocyte-derived neurotrophic factor (MANF), a kind of evolutionarily conserved neurotrophic factor. It was described few years ago as a survival promoting factor for embryonic midbrain dopaminergic neurons in vitro [1]. The protective effect of MANF is specific for dopaminergic neurons, and no effects on GABAergic or serotonergic neurons are detected [1].

Some studies have shown that MANF selectively protects dopaminergic neurons by inhibiting the neurotoxicity induced by unfolded protein response(UPR) [2, 3]. Additionally, MANF has been shown to regulate the endoplasmic reticulum stress (ER stress) induced gene transcription, such as HSP70 family, ATF family, xbp-1 and MAPK [4–6]. In our previous studies, we found that the expression level of HSP70 family, which is considered as a chaperon like heat shock protein family, play key roles in neuroprotection process. In SHSY-5Y cells treated with 6-OHDA or overexpressed α-synuclein, the expression of GRP78, a 78kd member of HSP70 family, is up-regulated under the treatment of MANF [7]. In PC12 cell line, the transcription level and protein level of HSP70 is up-regulated under 6-OHDA treatment [8]. GRP78 and HSP70 share a high degree of homology with HSP70 family [9]. They are considered to be involved in the survival pathway after ER stress [10]. Whether HSP70 is also involved in MANF signaling pathway is still unknown.

RNA sequencing (RNA-seq) is an unbiased sequencing tool that allows transcriptome profiling to detect gene expression changes in a cell or tissue sample. In this study, we aim to comprehensively investigate the expression level of ER stress relative genes in response to MANF treatment. For in vitro study, a DA neurons like cell line, SHSY-5Y cells [11], was employed and cell apoptosis was detected to verify the function of HSP70, which was found significantly changed in the transcriptome in response to MANF treatment. And we also found that MANF/HSP70 is 6-OHDA specific. Hydrogen peroxide solution (H_2_O_2_), tunicamycin (TM) and thapsigargin (TG) have been used as normal models for ER stress induced apoptosis. In TM, TG and H_2_O_2_-induced ER stress, the expression of HSP70 was not changed under MANF treatment.

## Methods

### SHSY-5Y cell culture

The human neuroblastoma cell line, SHSY-5Y, was cultured in DMEM/F-12 medium (life technologies, Carlsbad, CA, USA) containing L-Glutamine 300 mg/L, 10%FBS (fetal bovine serum) and kept at 37 °C in a humidified 5% CO2 incubator. 150 μM 6-OHDA (Sigma, St.Louis, MO) was used to induce apoptosis, leading to 50% reduction of viability after 24 h as measured with MTT assay.

### RNA-Seq

SHSY-5Y cells were harvested and total RNA was extracted using the TRIzol (Life Technologies) method. mRNA was further enriched from the total RNA using oligo dT magnetic beads (Life Technologies). Then, the mRNA was fragmented into short fragments (200–500 bp) using an RNA fragmentation kit (Ambion, Austin, TX). First strand cDNA was synthesized by random primers, followed by the synthesis of second strand cDNA. Double stranded cDNA was purified using a QIAquick PCR extraction kit (Qiagen, Hilden, Germany) and used for end repair and base A addition. For high throughput sequencing library constructed according to the manufacture’s instructions of BGI-Shenzhen (Beijing Genomics Institute, Shenzhen, China). Paired-end sequence reads were generated utilizing an illumina HiSeq 2000 instrument with TruSeq v3 reagents at BGI-Shenzhen.

### RNA Seq data analyses

For RNA Seq sequence reads, one base was trimmed from the 5’end and then the reads aligned to the human genome (hsa, NCBI) and transcriptome using TopHat [12]. The Refseq gene annotation file was provided for TopHat using the default parameters. Only uniquely and properly aligned read pairs were used for downstream analysis. The Cuffdiff program was used to calculate the relative abundance of each gene. A gene was defined to be differentially expressed with statistical significance if it met either of the following conditions: the fragments per kilobase of exon model per million fragments mapped (FPKM) value was zero in one sample and not less than 0.05 in the other, or the *q*-value calculated by Cuffdiff was less than 0.05. Gene ontology (GO) analysis was performed using the online program DAVID.

### Quantitative real-time PCR

To analyze HSP70 transcription by quantitative real-time PCR (qPCR), total RNA was extracted as described above and reverse-transcribed to cDNA using oligo-dT primer and Superscript II reverse transcriptase (Invitrogen, Darmstadt). The genetic expression of HSP70 was normalized to the internal reference control gene, β-actin. The following primer pairs were used: β-actin, forward primer: 5′- CCCAGATCATGTTTGAGACCT-3′ and reverse primer: 5′- CAGAGGCGTACAGGGATAGC-3′; human HSP70, forward primer: 5′- TAACCCCATCATCAGCGGAC -3′ and reverse primer: 5′-GAAGCTCCAAAACAAAAACAGCA -3′. human GRP78, forward primer:: 5′- AGACGGGCAAAGATGTCAGG -3’and reverse primer: 5′- GCCCGTTTGGCCTTTTCTAC -3′; human Xbp1, forward primer: 5′- CTGAGTCCGCAGCAGGTG -3’and reverse primer: 5′- TGTCCAGAATGCCCAACAGG -3′; human ATF4, forward primer: 5′- CTTGATGTCCCCCTTCGACC-3′, reverse primer: 5′- CTTGTCGCTGGAGAACCCAT-3′; human ATF6, forward primer: 5′- GAGTATTTTGTCCGCCTGCC -3′; reverse primer: 5′- GGCTCCCCCATTTCACAAGT -3′; human MAPK, forward primer: 5′- CAGGACTGCAGGAACGAGT -3′, Reverse primer: 5′- TTCCTTGTAGCCCATGCCAA -3′; GAPDH, forward primer:5′- GTCAAGGCTGAGAACGGGAA -3′, reverse primer:5′- TCGCCCCACTTGATTTTGGA -3′. The relative gene expression was calculated via the comparative Ct method.

### RNAi of HSP70

For hsp70 RNAi knockdown experiment in SH-SY5Y cells, we design the target site of small hairpin RNAs (shRNA) for hsp70 from 3476 to 3494 cDNA (NM_005347). 2 independent shRNAs (shHSP70–3 and shHSP70–4) is as follows. Synthesized shRNA template oligonucleotides were phosphorylated, annealed, and then ligated into linearized PLV-shRNA clotech vector digested with EcoRI/BamHI. SHSY-5Y were transfected with shRNA plasmid by Lipofectamine 3000 reagent (Invitrogen) and then were treated with drugs.

### Western blot analysis

Cells were homogenized in RIPA lysis buffer. Lysates were centrifuged at 14,000 rpm for 3 min, and protein concentrations in the supernatant were analyzed using a BCA Protein Quantitation Kit. Proteins (30 μg) were loaded to each lane and separated by electrophoresis on SDS–PAGE gels, followed by transferring to PVDF membranes. Membranes were incubated with 5% BSA for 1 h. Then, membranes were incubated with primary antibodies against hsp70 (4872; Cell Signaling Technology, USA), PERK(MA5–15033, Thermo Fisher, USA),caspase-3 (9579; Cell Signaling Technology, USA), and MANF (H00007873-M01; Abnova, USA), overnight at 4 °C. Membranes were incubated with horseradish-linked secondary antibodies (7074; Cell Signaling Technology, USA) for 2 h at 37 °C. Bands were visualized using an ECL detection system.

### TUNEL staining

TUNEL staining was performed according to manufacturer’s protocols. Briefly, cells were washed with PBS three times and fixed in 4% paraformaldehyde in PBS. Cells were treated with 50 μL of TUNEL reaction mixture for 1 h at 37 °C, then washed three times with PBS. Cells were counterstained with DAPI for 5 min and examined under a fluorescence microscope.

### Statistical analysis

All results were expressed as mean ± SEM. GraphPad Prism 5.0 software was used for statistical evaluation. Comparisons among different conditions were performed using one-way ANOVA and two-way ANOVA with Bonferroni post-test, and unpaired, two-tailed t test.

## Results

### 6-OHDA has neurotoxic effects on SHSY-5Y cells

**Fig. 1:**
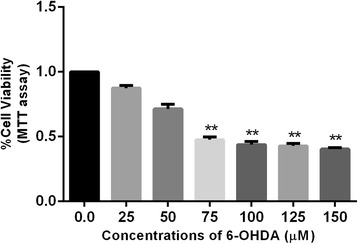
Assessment of 6-OHDA-induced toxicity in SHSY-5Y cells. Dose dependent neurotoxic effects of 6-OHDA in SHSY-5Y cells as detected by MTT assay. Values represent mean ± SEM, ***p < 0.001,*p < 0.05. One-way ANOVA

To induce excitotoxicity, SHSY-5Y cells were exposed to 6-OHDA in concentrations ranging from 25 to 150 μM for 24 h. A significant reduction in SHSY-5Y cell number was observed following incubation with 75-150 μM 6-OHDA, as determined by MTT assay (Fig. 1).

### MANF inhibits 6-OHDA-induced apoptosis in SHSY-5Y cells

**Fig. 2:**
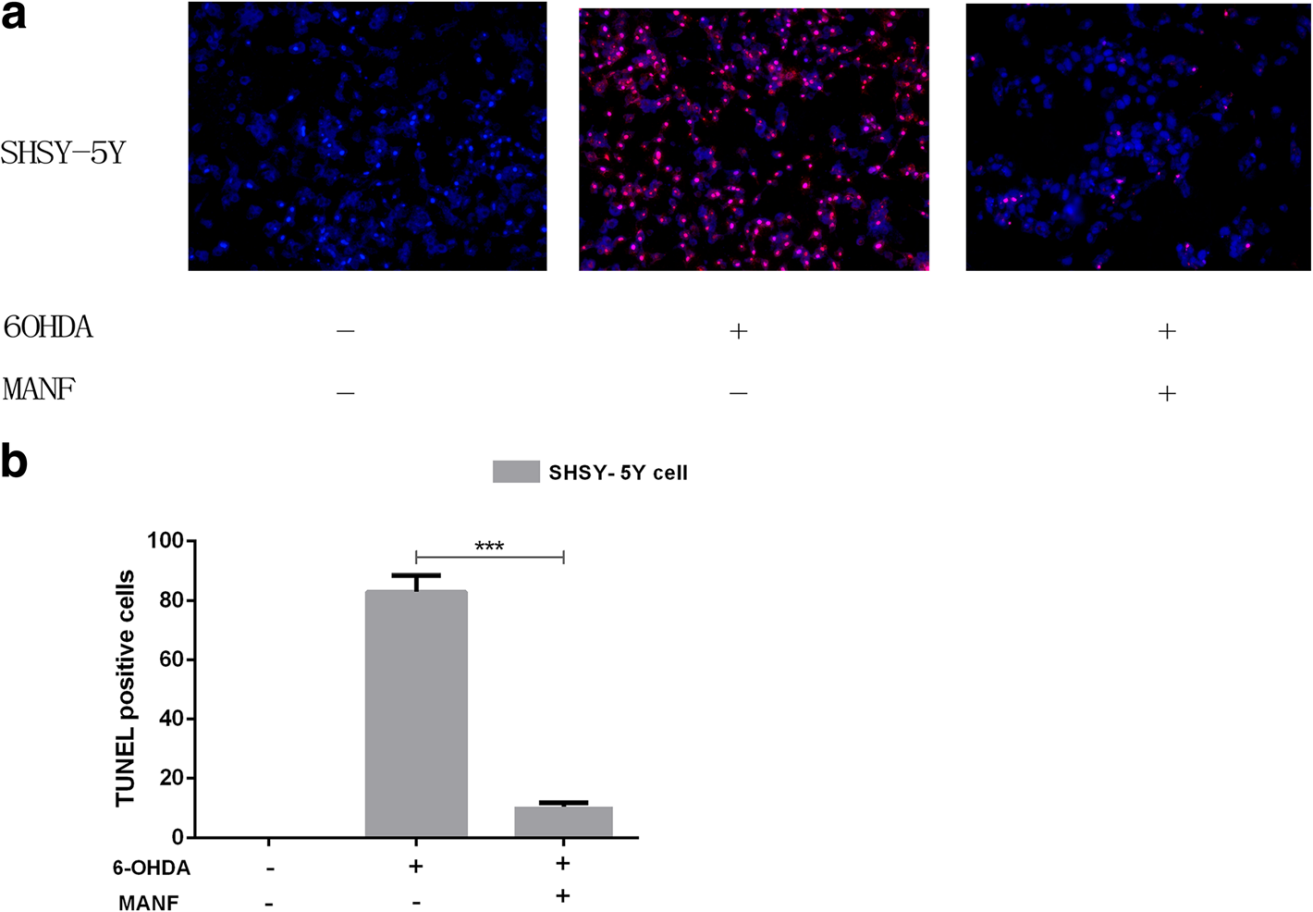
MANF inhibited 6-OHDA-induced apoptosis in SHSY-5Y cells. **a** Representative fluorescent images for DAPI and TUNEL merged images in control group; SHSY-5Y cells treated with 150 μM 6-OHDA; SHSY-5Y cells treated with 150 μM 6-OHDA + 4 μg/mL MANF; **b**: The treatment of MANF showed a significant protective effect against 6-OHDA toxicity. Values represent means ± SEM, ***p < 0.001, one-way ANOVA with Bonferroni post test

TUNEL staining significantly increased in SHSY-5Y cells treated with 6-OHDA, compared with control cells (Fig. 2a). Forty-eight hours of MANF (4 μg/ml) treatment significantly inhibited the increase in TUNEL staining induced by 6-OHDA treatment (Fig. 2a). These results suggest that MANF inhibited 6-OHDA-induced cell apoptosis. Cleaved caspase-3 level was significantly increased in SHSY-5Y cells under 6-OHDA treatment, compared with control cells (Fig. 6c). Forty-eight hours of MANF (4 μg) treatment significantly inhibited the increase in cleaved caspase-3 level induced by 6-OHDA (Fig. 6c). These results suggest that MANF inhibited 6-OHDA-induced cell apoptosis.

### Up-regulation of ER stress relative genes under MANF treatment

**Fig. 3:**
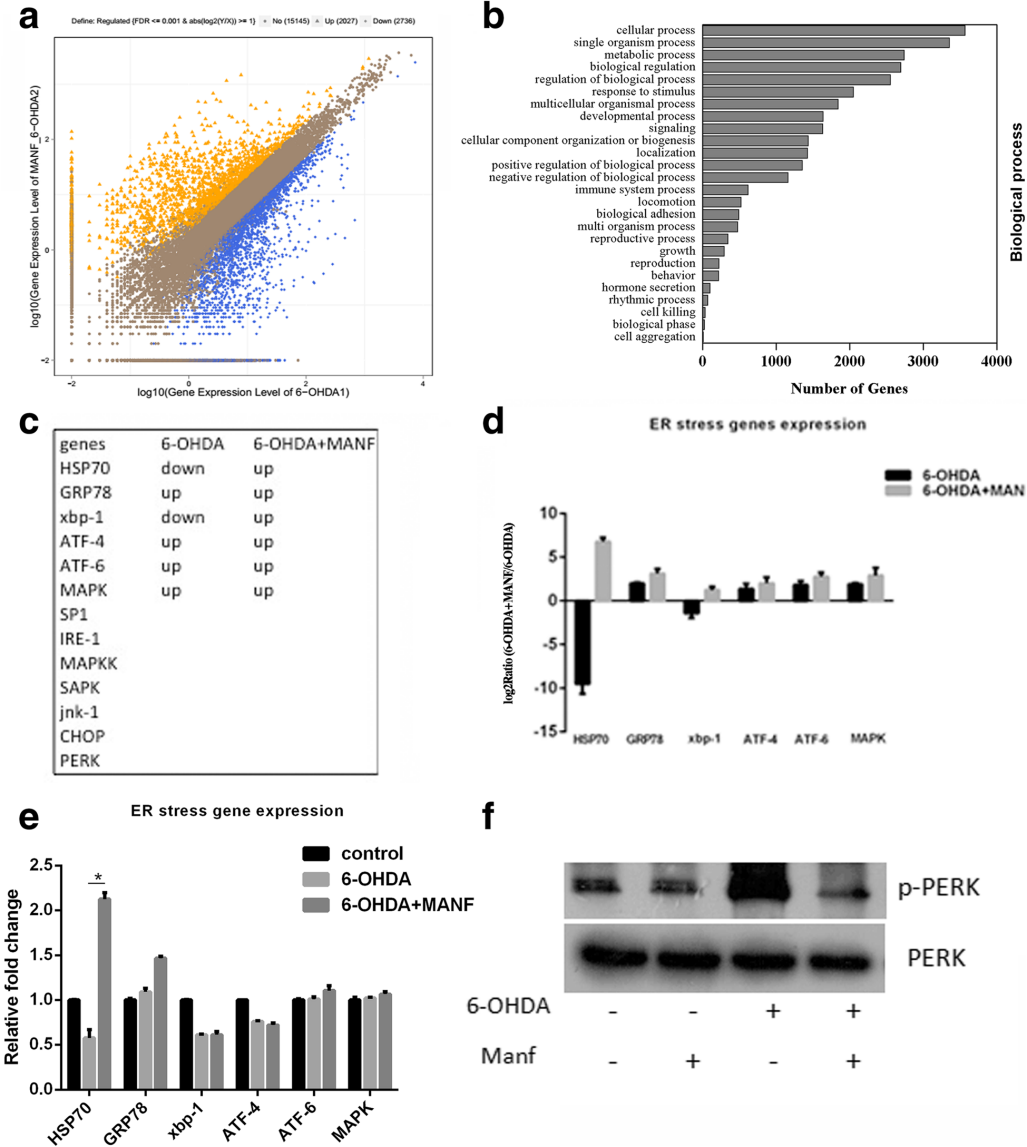
ER stress genes up-regulated in SHSY-5Y cells under MANF treatment. **a** Differential gene expression between 6-OHDA treatment group and 6-OHDA + MANF treatment group based on RNA-seq analyses, 2027 genes up-regulated; 2736 genes down-regulated; **b**: Gene ontology analysis of the top 26 biological processes associated with up regulated gene expression in the 6-OHDA + MANF treatment group relative to 6-OHDA treatment group (ranked by *q*-value). **c**, **d**: RNA-seq with whole gene profiling was performed to detect the differentially expressed genes in 6-OHDA + MANF cultures vs. 6-OHDA cultures in SHSY-5Y cells. These genes including HSP70, GRP78, MAPK, ATF6, ATF4 and xbp-1. **e**: Real-time PCR quantitation of HSP70, GRP78, xbp-1, ATF-4, ATF-6, MAPK mRNA levels in control, 6-OHDA and 6-OHDA + MANF treatment group (mean ± SEM, *n* = 5). The relative mRNA expression levels of HSP70, GRP78, xbp-1, ATF-4, ATF-6, MAPK in SHSY-5Y cells were normalized to those of GAPDH. (*p < 0.05; one-way ANOVA with Bonferroni post-tests). **f**: A representative Westernblot result shows the expression of phosphorylated PERK and PERK in control SHSY-5Y cells, SHSY-5Y cells treated with 250 μM of 6-OHDA; SHSY-5Y cells treated with 4 μg/mL of MANF; and SHSY-5Y cells treated with 250 μM 6-OHDA +4 μg/mL MANF at 5 min

To gain insight into the molecular mechanism underlying the protective effect of MANF against 6-OHDA-induced apoptosis, we performed whole transcriptome profiling using RNA-seq. For each condition (control, MANF, 6-OHDA, 6-OHDA + MANF), total RNA was isolated. Then, mRNA was further purified and processed for next-generation sequencing. The difference in the read coverage of gene expression between the *6-OHDA* and *6-OHDA + MANF* is shown in Fig. 3a. These analyses identified 4763 genes with statistically significant differences in gene expression levels between the two samples; 2027 genes were up-regulated and 2736 were down-regulated in 6-OHDA + MANF group relative to the 6-OHDA group. GO analysis showed that the up-regulated genes in the *6-OHDA + MANF* samples were enriched in cellular process (Fig. 3b). Strikingly, two ER stress genes (HSP70, xbp-1) were down-regulated and four ER stress genes (GRP78, ATF4, ATF6, MAPK) were up-regulated under 6-OHDA treatment. Six ER stress relative genes (HSP70, GRP78, xbp-1, ATF-4, ATF-6, MAPK) were found to be enriched in 6-OHDA + MANF treatment group(Fig. 3c、d). HSP70 was the most significantly up-regulated gene under 6-OHDA + MANF treatment in SHSY-5Y cells. The expression of the other ER stress relative genes (SP1, IRE-1, MAPKK, SAPK, jnk-1, CHOP, PERK) was not changed under 6-OHDA + MANF treatment (Fig. 3c). To verify the differential expression of the ER stress genes in *6-OHDA* and *6-OHDA + MANF* group, qPCR was employed to measure the relative abundance of mRNA. To validate the gene expression data obtained from RNA-seq, reverse transcription-quantitative polymerase chain reaction (RT-PCR) was performed. Six changes were selected based on involvement in the ER stress pathway (HSP70, GRP78, xbp-1, ATF-4, ATF-6 and MAPK). RT-PCR confirmed the significant changes in mRNA expression that was observed by RNA-seq after 24 h of 6-OHDA + MANF treatment (Fig. 3e). Phosphorylated PERK was checked by western blot. The reaction of kinase is short, so it can be seen in 5 min after injury. At the time point of 5 mins, the samples under the treatment of 6-OHDA + MANF, phosphorylated PERK did not increase, indicating the protective effect of MANF (Fig. 3f).

### MANF up-regulates HSP70 expression in SHSY-5Y cells treated with 6-OHDA

**Fig. 4:**
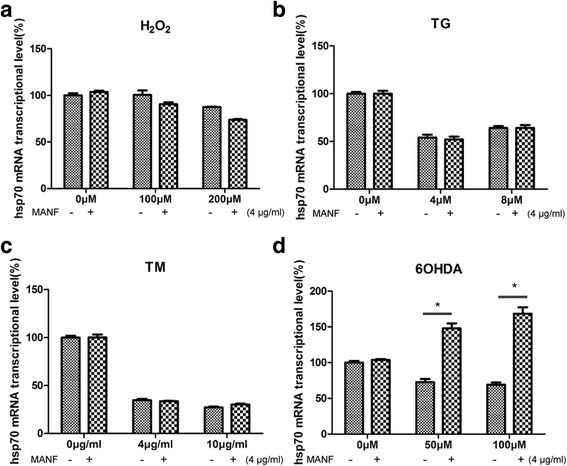
MANF did not upregulate HSP70 expression levels under H_2_O_2_ TG and TM treatment in SHSY-5Y cells. **a** Real-time PCR quantitation of HSP70 mRNA levels in control, H_2_O_2_ and H_2_O_2_ + MANF treatment group (mean ± SEM, *n* = 5). The relative mRNA expression levels of HSP70 in SHSY-5Y cells were normalized to those of β-actin. **b** Real-time PCR quantitation of HSP70 mRNA levels in control, TG and TG + MANF treatment group (mean ± SEM, *n* = 5). **c** Real-time PCR quantitation of HSP70 mRNA levels in control, TM and TM + MANF treatment group (mean ± SEM, *n* = 5). **d** Real-time PCR quantitation of HSP70 mRNA levels in control, 6-OHDA and 6-OHDA + MANF treatment group (mean ± SEM, *n* = 5). The relative mRNA expression levels of HSP70 in SHSY-5Y cells were normalized to those of β-actin

In the ER stress genes, HSP70 was the most significantly up-regulated gene. We investigated the effect of MANF on HSP70 expressions in SHSY-5Y cells treated with 6-OHDA. HSP70 expression levels did significantly decrease in cells treated with 6-OHDA compared with control cells. MANF treatment resulted in a significant increase in HSP70 expression in 6-OHDA-treated cells (*P < 0.05; Fig. 3d). The observed expression of log2 Ratio (MANF + 6-OHDA/6-OHDA) for HSP70 was significantly increased (fold change = 7.07) in SHSY-5Y cells of *6-OHDA + MANF* treatment group. To verify the differential expression of HSP70 in *6-OHDA* and *6-OHDA + MANF* group, qPCR was employed to measure the relative abundance of HSP70 mRNA. Compared with *6-OHDA* group, the SHSY-5Y cells in *6-OHDA + MANF* group exhibited 2.15-fold increase in HSP70 transcription, as determined by qPCR (Fig. 3e), which is consistent with the 7.07-fold increase observed by RNA-Seq analysis (Fig. 3c, d). But HSP70 expression did not significantly increase under H_2_O_2_ + MANF, TG + MANF and TM + MANF treatment in SHSY-5Y cells than in control cells (Fig. 4).

### RNAi knockdown for HSP70 abolishes the MANF protective effect against 6-OHDA-induced apoptosis in SHSY-5Y cells

**Fig. 5:**
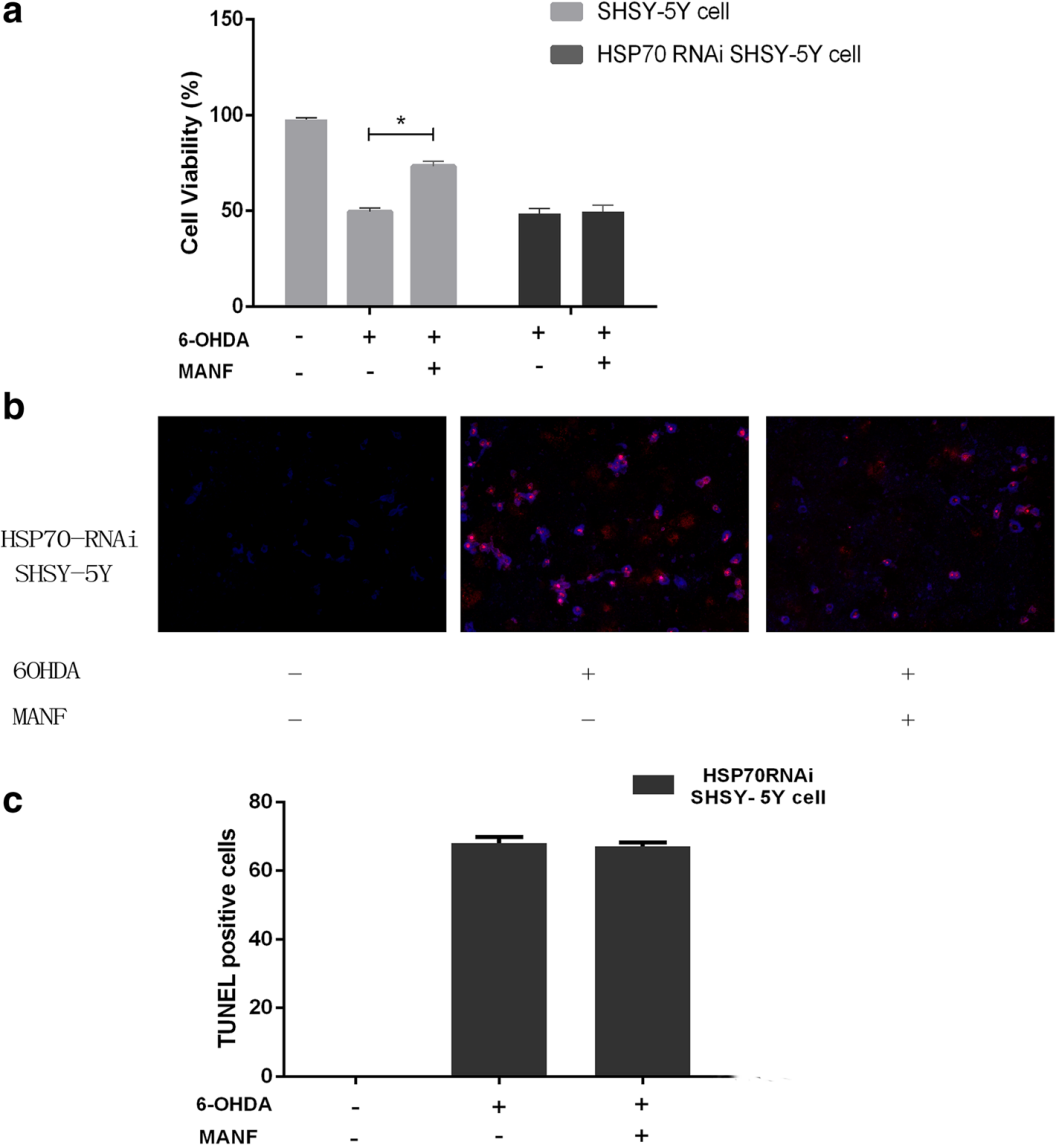
Effects of HSP70 blockade on the protective effects of MANF against 6-OHDA induced apoptosis in SHSY-5Y cells. **a** SHSY-5Y cells were pretreated with control RNAi of HSP70 for 48 h and then stimulated with 150 μM 6-OHDA or 150 μM 6-OHDA + 4 μg/mL MANF for further 48 h. Cell viability was measured by MTT. (*p < 0.05; one-way ANOVA with Bonferroni post-tests). **b** Representative fluorescent images for DAPI and TUNEL merged images in control group HSP70 RNAi SHSY-5Y cells; HSP70 RNAi SHSY-5Y cells treated with 150 μM 6-OHDA; HSP70 RNAi SHSY-5Y cells treated with 150 μM 6-OHDA +4 μg/mL MANF; scale bar, 100 μm. **c** The protective effect of MANF against 6-OHDA-induced apoptosis disappeared in HSP70 RNAi SHSH-5Y cells

**Fig. 6:**
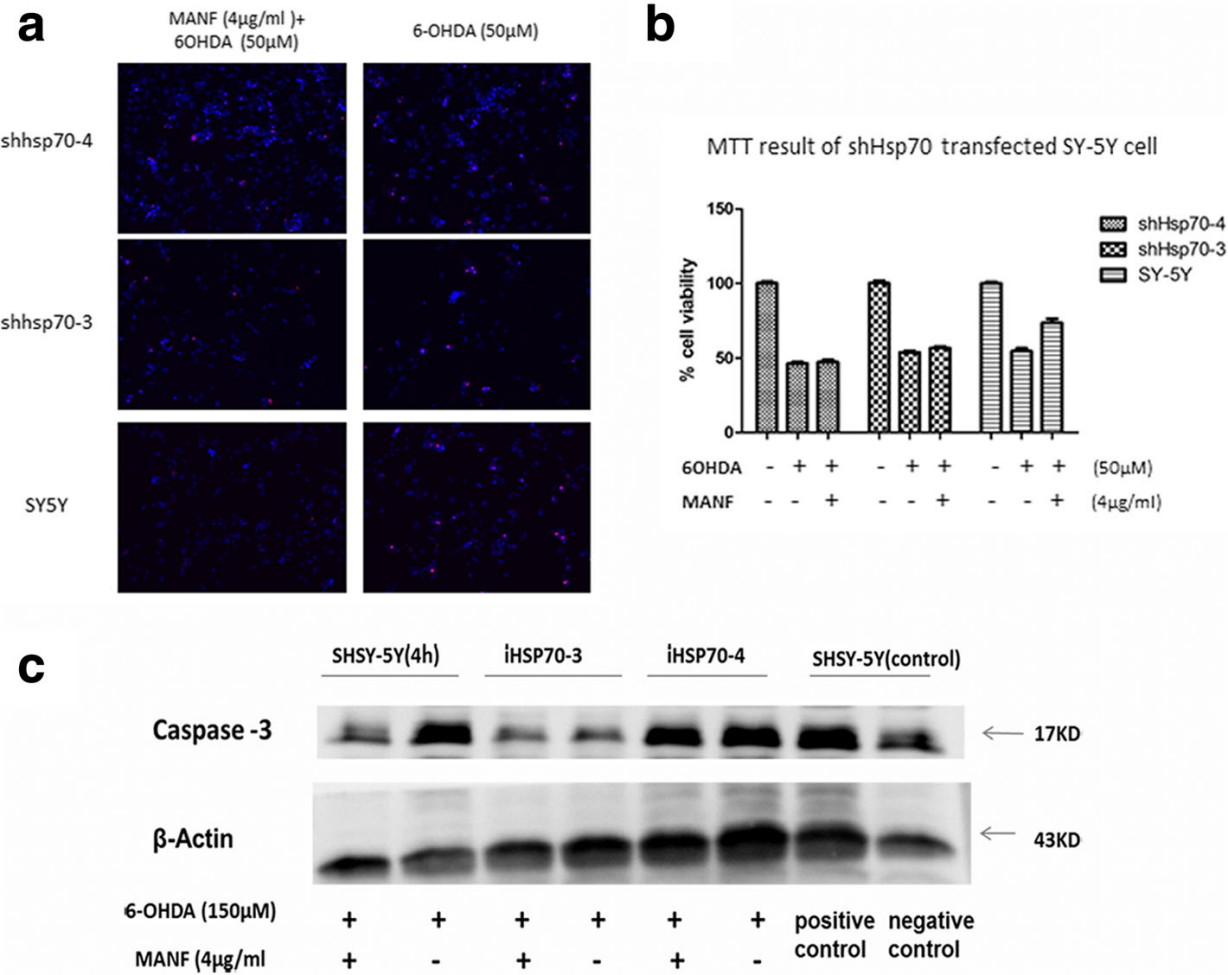
Effects of shHSP70–3/−4 blockade on the protective effects of MANF against 6-OHDA induced apoptosis in SHSY-5Y cells. **a, b** SHSY-5Y cells were pretreated with control RNAi of shHSP70–3/−4 for 48 h and then stimulated with 50 μM 6-OHDA or 50 μM 6-OHDA + 4 μg/mL MANF for further 48 h. Cell viability was measured by TUNEL and MTT. **c** A representative Western blot result shows the expression of cleaved caspase-3 in control SHSY-5Y cells, SHSY-5Y cells treated with 150 μM 6-OHDA +4 μg/mL of MANF; 150 μM of 6-OHDA; A representative Western blot result shows the expression of cleaved caspase-3 in HSP70–3/−4RNAi SHSY-5Y cells treated with 150 μM 6-OHDA +4 μg/mL of MANF and 150 μM of 6-OHDA. (*p < 0.05; one-way ANOVA with Bonferroni post-tests)

Several lines of evidence have shown that HSP70 protects cells from 6-OHDA-induced cell apoptosis. We showed that RNAi knockdown for HSP70 could block MANF-induced survival of SHSY-5Y cells. These results suggest that HSP70 inhibited 6-OHDA-induced apoptosis in SHSY-5Y cells (Fig. 5a). Similarly, TUNEL staining induced by 6-OHDA treatment did not decrease under MANF treatment in HSP70 RNAi SHSY-5Y cells (Fig. 5b, c). In parallel with this result, we found that in HSP70 RNAi SHSY-5Y cells, MANF (4 μg) treatment did not significantly inhibit the increase in cleaved caspase-3 level induced by 6-OHDA (Fig. 6c). And two independent shRNAs (shHSP70–3 and shHSP70–4) were knocked down and knocking down efficiency was 0.32 and 0.22. We found that in both shHSP70–3 and shHSP70–4 RNAi SHSY-5Y cells, the protective effect of MANF against 6-OHDA induced apoptosis disappeared. TUNEL staining induced by 6-OHDA treatment did not decrease under MANF treatment in shHSP70–3/−4 RNAi SHSY-5Y cells (Fig. 6a). In MTT assay, we did not observe that the protective effect of MANF in shHSP70–3/−4 RNAi SHSY-5Y cells (Fig. 6b).

## Discussion

Recently, MANF has been recognized as an important factor that protect dopaminergic neurons in the substantia nigra from injury induced by several toxins [13]. In this study, we found that MANF inhibited 6-OHDA-induced apoptosis in SHSY-5Y cells. 6-OHDA is a neurotoxin widely used to create animal models of PD. In the experiment, the expression of TUNEL staining was observed by immunohistochemical staining to determine the number of apoptotic cells. We found that 6-OHDA increased the positive TUNEL staining, a marker of apoptosis. MANF treatment inhibited the increase of TUNEL staining induced by 6-OHDA. Our findings are consistent with recent studies, showing that MANF inhibits ER stress relative apoptosis [14]. That means, the protective effect of MANF were due to ER stress relative apoptosis suppression.

Our present understanding of the neuron protection mechanism of MANF suggests that it is involved in the regulation of ER stress and unfolded protein response (UPR) [3, 15]. In our study, expression of two ER stress marker genes, HSP70 and GRP78 were up-regulated under MANF + 6-OHDA treatment. This is in parallel with previous studies which showed that MANF reduces ER stress and inhibits ER stress-induced neuronal apoptosis [16]. We identified that MANF treatment significantly up-regulated HSP70 levels in SHSY-5Y cell models, which was accompanied by a decrease in cleaved caspase-3 level, suggesting that MANF alleviates ER-induced apoptosis through HSP70 up-regulation. Lee et al. demonstrated that MANF was an ER stress-responsive gene. MANF mRNA and protein were induced on ATF6 activation in the myocardium, in vivo*,* consistent with the hypothesis that ER stresses, such as ischemia, which can activate ATF6, a hallmark of ER stress genes, might also induce MANF expression in the heart [17, 18]. Rescently, it has been reported that lack of MANF in vivo in mouse leads to chronic activation of UPR [3]. ER stress and chronic activation of UPR play an important role in the pathogenesis of PD [19]. Thus, MANF has significant potential as a treatment of PD.

In a whole-genome microarray and pathway analysis, the expression of genes involved in membrane transport and the ER stress was found to be altered, in the absence of MANF, in drosophilaa models [20]. In parallel with this study, our RNA-seq result revealed that many of the genes up-regulated in MANF treatment group were associated with biological processes/pathways related to stimulus response. In ER stress relative genes, HSP70 and Grp78 changed significantly. Combined with our previous work, we found they were both involved in a survival signal transduction. Jessie S et al. showed that after a transient ischemic insult, the subcellular responses to the accumulation of unfolded proteins varies between cellular compartments and are most prevalent in the cytoplasm and, to a lesser degree, in the mitochondrial matrix and ER lumen. Induction of mRNA for HSP70 occurred earlier (beginning at 30 min) and more moderate (4-24 h) induction of mRNAs for ER lumen Grp78 [21]. Grp78 up-regulation is associated with HSP70-HSP90 client proteins [22]. Cellular events can trigger the unfolded protein response (UPR) and activate the expression of a number of genes involved in pro-survival pathways. Whether the HSP70 and Grp78 participate in a same signal cascade or belong to individual signaling pathways still needs to be investigated. However, aside from HSP70, no other anti-protein misfolding genes were differentially expressed in 6-OHDA + MANF treatment group, suggesting that up-regulation of HSP70 could be responsible for the protective effects of MANF in pathogenic condition of PD. Our Real time-PCR result could further show an increase in HSP70 levels in SHSY-5Y cells under MANF treatment. Similarly, in this study, we found that HSP70 knockdown inhibited the decrease in active caspase-3 levels induced by 6-OHDA, suggesting that HSP70 up-regulation may protect cells from 6-OHDA-induced apoptosis.

Various lines of evidence indicate that HSP70 siRNA transfection can further induce cell death in neuronal cells exposed to 6-OHDA [23, 24]. In our study, we showed that RNAi knockdown for HSP70 could block the protection of MANF against 6-OHDA induced apoptosis. In this research, we also observed that 6-OHDA treatment leads to suppression of HSP70 in SHSY-5Y cells. This result is on the contrary to the evidence in PC12 cells, in which 6-OHDA up-regulates the expression of HSP70. This may be due to PC12 cells and SHSY-5Y cells derived from different organs [23, 24].

HSP70 is a member of heat shock protein family, and it plays a critical function in the rescue of misfolded proteins [25–27]. HSP70-related anti-fibrillation may have contributed to the protection of dopaminergic neurons in MANF-treated cells and PD animals [28]. Earlier studies have shown that the aggregation ofα-Synuclein is linked to the pathogenesis of PD. Indeed, α-Syn is the major component of Lewy bodies. Molecular chaperones, which could modulate the pathological conversion of misfolded proteins into cytotoxic species, are recognized as key players in the avoidance of misfolding proteins [29–31]. The family of stress-inducible 70KDa heat-shock proteins (HSP70s), plays a critical function in the rescue of misfolded proteins [25–27], hence avoiding the potentially harmful effects of the aggregation of the species. Meanwhile, some studies showed that the expression of HSP70 was highly perturbed in the substantia nigra of PD patients, which was the site of neurodegeneration in this condition [32–34]. And in our H_2_O_2_, TG and TM system, the relationship between MANF and HSP70 was not observed. That means MANF/HSP70 was specific to 6-OHDA induced apoptosis. TM is a bacterial toxin that inhibits N-linked glycosylation of nascent proteins resulting in activation of UPR in mammalian cells [35]. TG has the ability to activate extracellular-signal regulated kinase (ERK), but tunicamycin does not [36]. It is well known that thapsigargin induces ER stress by blocking sarco-endoplasmic reticulum Ca^2+^-ATPase [36]. H_2_O_2_ is a representative ROS that is generated from nearly all sources of oxidative stress and can diffuse freely in and out of tissues [37]. The cytotoxic mechanisms are different between 6-OHDA and tunicamycin. 6-OHDA increases and accumulates reactive oxygen species (ROS) to induce ER stress; however, tunicamycin blocks N-linked glycosylation, accumulating unfolded proteins that induce ER stress [38]. This may explain MANF/HSP70 was specific to 6-OHDA.

## Conclusions

Our study indicates that MANF exerts a protective role against in vitro induced apoptosis in SHSY-5Y cells. This function may involve the activation of apoptosis inhibition. The regulation of HSP70 expression contributes to MANF-mediated neuroprotection. It is crucial to highlight and further characterize disease-related differences in basal and inducible gene expression levels. Further studies need to specifically define potential disease-related alterations in the reactivity to MANF and further define which molecular pathway is involved. Our results support the potential of MANF to contribute to a more protective environment for degenerating dopaminergic neurons in PD via regulation of HSP70 and cytokine secretion and therefore to be further evaluated as novel therapeutic approach for the treatment of PD.

## Abbreviations

6-OHDA: 6-hydroxydopamine
ER: Endoplasmic reticulum
HSP70: Heat shock protein
MANF: Mesencephalic astrocyte-derived neurotrophic factor
PD: Parkinson’s disease
